# Reconditioning Degraded Mine Site Soils With Exogenous Soil Microbes: Plant Fitness and Soil Microbiome Outcomes

**DOI:** 10.3389/fmicb.2019.01617

**Published:** 2019-07-10

**Authors:** Benjamin Moreira-Grez, Miriam Muñoz-Rojas, Khalil Kariman, Paul Storer, Anthony G. O’Donnell, Deepak Kumaresan, Andrew S. Whiteley

**Affiliations:** ^1^UWA School of Agriculture and Environment, The University of Western Australia, Perth, WA, Australia; ^2^Centre for Ecosystem Science, School of Biological, Earth and Environmental Sciences, University of New South Wales, Sydney, NSW, Australia; ^3^School of Biological Sciences, University of Western Australia, Perth, WA, Australia; ^4^Kings Park Science, Department of Biodiversity, Conservation and Attractions, Perth, WA, Australia; ^5^Troforte Innovations Pty Ltd., Perth, WA, Australia; ^6^Faculty of Science, University of Western Australia, Perth, WA, Australia; ^7^School of Biological Sciences, Queen’s University of Belfast, Belfast, United Kingdom

**Keywords:** arid zone, mine site restoration, microbiome diversity, soil inocula amendments, soil microbiome

## Abstract

Mining of mineral resources substantially alters both the above and below-ground soil ecosystem, which then requires rehabilitation back to a pre-mining state. For belowground rehabilitation, recovery of the soil microbiome to a state which can support key biogeochemical cycles, and effective plant colonization is usually required. One solution proposed has been to translate microbial inocula from agricultural systems to mine rehabilitation scenarios, as a means of reconditioning the soil microbiome for planting. Here, we experimentally determine both the aboveground plant fitness outcomes and belowground soil microbiome effects of a commercially available soil microbial inocula (SMI). We analyzed treatment effects at four levels of complexity; no SMI addition control, Nitrogen addition alone, SMI addition and SMI plus Nitrogen addition over a 12-week period. Our culture independent analyses indicated that SMIs had a differential response over the 12-week incubation period, where only a small number of the consortium members persisted in the semi-arid ecosystem, and generated variable plant fitness responses, likely due to plant-microbiome physiological mismatching and low survival rates of many of the SMI constituents. We suggest that new developments in custom-made SMIs to increase rehabilitation success in mine site restoration are required, primarily based upon the need for SMIs to be ecologically adapted to both the prevailing edaphic conditions and a wide range of plant species likely to be encountered.

## Introduction

The mining of ores and minerals results in deleterious environmental outcomes (Bradshaw, 2000) such as clearance of landscape biota and the production of large amount of by-products. Central to the amelioration of mining impacts is the rehabilitation of mined landscapes, with the final goal of the establishment of aboveground flora and fauna of adequate composition, and diversity (Maestre et al., 2005; Baldwin et al., 2006; Brooker, 2006). In parallel, the belowground microbiome also needs to be rehabilitated, but is often overlooked, a key goal being the generation of high microbial diversity which can produce stable ecosystem services. These services are critical since they have central roles in the supply to the plant of key nutrients, pathogen protection, and water access. Therefore, in order to maximize the outcomes from aboveground restoration approaches we need to develop equivalent belowground microbiome “reconditioning” strategies which provide an optimum soil microbiome, one that will sustain aboveground biomass, and provision long term seed emergence and survival.

During mining operations, initial vegetation clearance is followed by removal, and storage of the topsoil to expose the deeper mineral containing substrates. This topsoil is usually stored in a non-planted state for extended periods of time, often years. Over time, there are significant declines in key traits such as carbon content, seed banks of locally adapted native plants, and the composition of the microbiome (Golos and Dixon, 2014; Muñoz-Rojas et al., 2016a), including community diversity, and function (Kumaresan et al., 2017). This decline in the soil microbiome is a critical factor within this stored topsoil, not only due to potential loss of key nutrient cycling pathways (Wagg et al., 2014) but growing evidence also suggests that plant diversity, and fitness can be determined by the surrounding microbiome composition (Lau and Lennon, 2011, 2012; Panke-Buisse et al., 2015; Wubs et al., 2016). To this end, the addition of exogenous microbiomes in the form of soil microbial inocula (SMIs), such as those traditionally used in agricultural practices, is becoming increasingly mainstream in mine site restoration practices.

Due to the economic importance of agriculture (Johnston and Mellor, 1961; Knowler and Bradshaw, 2007) SMIs have been developed over decades for addition to the soil to maximize plant establishment, growth and productivity. These include plant growth promoting rhizobacteria (PGPR) developed and deployed across a range of agricultural soil types (Çakmakçi et al., 2006; Singh et al., 2011; Chaparro et al., 2012; Upadhyay et al., 2012). Broadly, PGPRs encompass Nitrogen fixation and P solubilizing microorganisms such as *Rhizobium*, *Pseudomonas*, *Azotobacter*, and *Azospirillum* which directly, or indirectly, promote plant growth (Singh et al., 2011). Additionally, inoculation with microorganisms such as *Trichoderma*, *Pseudomonas*, *Glomus*, *Bacillus*, and *Agrobacterium* can alter the plant’s physiological state and enhance plant growth (Conrath et al., 2006; Rodrigues et al., 2008) and response to environmental stress (Jung et al., 2012). The addition of microorganisms to trigger such processes is termed “bio-priming” and has been successfully applied to increase production in wheat (Meena et al., 2016), rice (Rodrigues et al., 2008), maize (Akladious and Abbas, 2012), and soybean (Entesari et al., 2013). Finally, the supplementation of SMIs to mineral fertilizers to allow slow and controlled release of nutrients has gained increased attention as a means of efficient nutrient use and has shown significant promise in improving crop growth (Wu et al., 2005; Chang and Yang, 2009; Pereira et al., 2009; Leaungvutiviroj et al., 2010).

Whilst important and clearly beneficial in agricultural systems, the ecological outcomes of deploying SMIs to mine site systems have been little studied. One recent example has shown the successful increase in germination and seedling growth of two plant species native to Western Australia, using indigenous cyanobacteria isolates (Muñoz-Rojas et al., 2018). However, there is significant interest in using existing commercially produced SMIs to directly apply the technology within a mine site restoration setting. Theoretically, several barriers may exist to their effective deployment. These include much lower nascent nutrient levels in mine soil substrates, which will dictate whether copiotroph or oligotrophic adapted microorganisms survive and drive the ecosystem functionality (Fierer et al., 2007; Carbonetto et al., 2014) and the ratio of bacteria to fungi, an important ecosystem property (Güsewell and Gessner, 2009). Further, natural ecosystems tend to harbor microbiome diversity which has co-evolved with the native plants, including taxa that promote germination (Muñoz-Rojas et al., 2018), or those that can solubilize recalcitrant macronutrients (Lambers et al., 2009).

Here, in order to resolve the efficacy of agricultural derived SMIs within mine site rehabilitation strategies, we assess the influence of an agricultural derived SMI upon the fitness of an Australian native plant *Acacia ancistrocarpa*, dominant in arid lands in Western Australia and commonly used for restoring semi-arid mine sites. We determined seedling emergence, a critical life stage transition in arid plants, and shoot to root ratio in tandem with emergent properties of the soil microbiomes in the first effort to assess the potential rehabilitation implication of an SMI consortium that is used in agriculture. We conclude that agricultural derived SMIs can be compromised in habitats such as semi-arid ecosystems and SMIs derived from cognate environments are likely have a higher chance of efficacy and plant growth promotion.

## Materials and Methods

### Experimental Design and Set Up

This study was conducted between December 2015 and February 2016 in the glasshouse facilities located at Kings Park and Botanic Garden in Perth, Western Australia. *A. ancistrocarpa*, a nitrogen-fixing legume native to the Pilbara and other regions of Western Australia, and commonly used in arid zone restoration (Bateman et al., 2016) was selected for this experiment. Topsoil retrieved from previously stockpiled material (approximately the top 10– 20 cm of the soil profile) was collected from an active mine site in the southern part of the Pilbara region (23°21′14″S 119°43′55″E), transported to the Kings Park glasshouse facilities in 200L drums and used as growth media (Muñoz-Rojas et al., 2016b; Kneller et al., 2018). The experimental design consisted of four treatments, all derived from a parent soil (Basal), consisting of an incubated control with no amendments (Control); nutrient addition alone in the form of ammonium sulfate [(NH_4_)_2_SO_4_] added at 150 mg N (Nitrogen), a commercially available SMI added at 0.2 g/100g^–1^ soil (Microbes) and both nitrogen and microbial consortia (Microbes+Nitrogen). This allowed the experimental determination of responses above control for a standard nutrient amendment, an SMI amendment and potential additive effect of both. Pots of 25 cm^2^ surface by 12 cm height were assorted in a randomized block design and replicated 10 times. Ten seeds, previously treated during 1–2 min in hot water at 90°C to break physical dormancy (Erickson et al., 2016) were sown into each pot. Pots were maintained at field capacity) throughout the experiment. After 12 weeks, plants were harvested, and bulk soil was taken for analyses.

### Analysis of Soil Chemical and Biological Characteristics and Plant Parameters

At the end of the experiment, three soil samples of 150 g were collected from three randomly selected replicates from each treatment. Additionally, three soil samples (of 150 g) were taken from the topsoil drum used for the experiment in order to assess the baseline microbial configuration (Basal treatments). Three extra replicates were amended with SMI at the same concentration used for the soil incubations (inoculum soil) in order to identify possible changes in the soil chemistry composition by carrier compounds that might be present in the soil microbial inoculum. Soil samples were sub-divided into two; one subsample was air-dried and sieved (2 mm) for chemical analysis, and the other was immediately taken to the lab and analyzed for microbial activity and DNA extraction. Soil microbial activity (ppm-CO_2_) was measured using the 1-day CO_2_ Solvita test which determines soil microbial respiration rate based on the measurement of the CO_2_ burst produced after moistening dry soil and incubation at 25°C for 24 h (Muñoz-Rojas et al., 2016a). All other soil chemical parameter including organic Carbon (Org.C), nitrogen (as in NH_4_-N and NO_x_-N), Sulfur (S), electrical conductivity (EC), pH-CaCl_2_, Copper (Cu), Iron (Fe), Manganese (Mn), Zinc (Zn), Aluminum-exc (ex.Al), Calcium-exc (ex.Ca), Magnesium-exc (ex.Mg), Potassium-exc (ex.K) and Sodium-exc (ex.Na) were characterized through CSBP Plant and Soil Laboratory (Bibra Lake, Western Australia).

Seedling emergence (%) was determined as the average seedlings that emerged per pot after 16 days divided by the number of seeds sown per pot (Muñoz-Rojas et al., 2016b; Kneller et al., 2018). To determine plant growth parameters (seedling shoot and root length and biomass), plant materials (one random plant per pot, *n* = 4) were harvested after 12 weeks and assessed as in Bateman et al. (2016). Root and shoot lengths were measured using a flatbed scanner (WinRHIZO, Regent Instruments, Sainte Foy, Canada). Subsequently plants were dried at 75°C for 72 h and weighed using a five-point balance PB403-S/FACT to determine total biomass.

### DNA Extraction, PCR Amplification, and Bioinformatic Analysis

DNA was extracted in triplicate from 0.25 g of soil samples using a Powersoil-htp96 Soil DNA isolation kit (MO BIO Laboratories, CA, United States) following manufacturer’s guidelines with minor modifications [freeze-thaw cycle (x3) after the addition of solution C1]. Extracted DNA was quantified using a QUBIT2.0 fluorometer (Life Technologies, United States) and 2 ng of DNA was used as template for subsequent PCR amplification using 515F/806R primer set (targeting the 16S rRNA V4 region for both bacteria and archaeal domains) (Liu et al., 2007). See Kumaresan et al. (2017) for detailed PCR reagent and thermal conditions. PCR amplicons were checked for both length and specificity, purified using AMPure (Beckman Coulter, Australia), blended into an equimolar pool, and sequenced using an Ion Torrent PGM platform (Thermo Fisher Scientific).

All bioinformatic analysis were performed within the QIIME platform (Caporaso et al., 2010). Briefly, 1150283 raw reads were filtered (minimum average quality = 20, maximum/minimum sequence length = 350/130, respectively, no primer mismatch or barcode error allowed, maximum length of homopolymers = 15 and maximum number of ambiguous bases = 6). 193347 filtered sequences were further checked for chimeric sequences using the USEARCH algorithm (ver 6.1) rejecting ∼11.9%. The remaining 177074 high quality reads were then subjected to *de novo* OTU picking at 97% sequence identity using UCLUST (ver1.2.22q) and taxonomy was assigned using the RDP classifier (Wang et al., 2007) using the Greengenes database (ver 13.8) (DeSantis et al., 2006). The sequences for each sample were rarefied at a depth of 2700 reads for statistical analyses and all amplicon sequences associated with this article were deposited in ENA under the project accession number PRJEB25854.

### Statistical Analysis

One-way analysis of similarity (ANOSIM) was performed to test any differences between soil chemistry across treatments. The pairwise *R* values output matrix was then used to compute non-metric multi-dimensional scaling (n-MDS). n-MDS plots and R distribution histograms were plotted in R using the ggplot2 package (Wickham, 2009). Soil microbial activity and plant growth parameters were tested for normality and variance homogeneity using the Shapiro–Wilk and Levene tests, and these data were log transformed as necessary. Differences in variables among treatments were tested using one-way analysis of variance (ANOVA) and comparisons between means were performed with Tukey’s HSD (*P* ≤ 0.05). Diversity measures (Shannon diversity and evenness) were calculated using the function “*diversity*” available through the Vegan package (Oksanen et al., 2017) within R (R Core Team, 2015). Boxplot and heatmaps were computed using ggplot2 and pheatmap (Kolde, 2015) libraries, based upon OTU tables at order level. Prior to heatmap generation, the OTU table was fourth transformed and filtered to include only the top-20 most abundant taxonomic bins presented within Treatment Inoculum, to identify and track changes within the microbial consortia used through the soil incubations. Correspondence analysis was performed using the function *cca* within Vegan and plotted using ggvegan (Simpson, 2015) and ggrepel (Slowikowski, 2016) libraries. In order to graphically assess similarities between microbial composition and chemical characteristics, cluster analysis and SIMPROF test were performed within Primer-E 7 (Clarke and Gorley, 2015) based on a Bray-Curtis and Euclidean distance matrices for amplicon sequencing and soil chemistry, respectively. Pairwise OTUs similarities were computed using the script *shared_phylotypes.py* available within QIIME 1.9. All figures were edited in Inkscape, a freely available vector suite (https://inkscape.org/).

## Results

### Soil Chemical Properties

**FIGURE 1:**
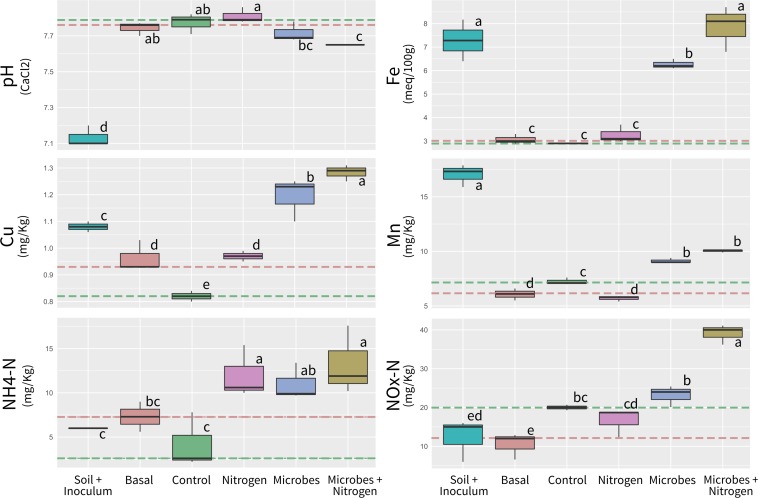
Boxplot showing main soil chemistry parameters measured in the study. Orange and green segmented lines represent Basal (previous incubation) and Control (after incubation) levels. Boxplot sharing same letter coding are significantly similar (*p* ≤ 0.05).

In order to identify possible changes is the soil chemistry solely due to adding exogenous compounds (such as the microbial biomass and any carrier molecules within the SMI preparation), soil chemistry analyses were performed upon native topsoil amended with the SMI (Inoculum) at the concentrations used to initiate the incubations. Furthermore, analyses were also performed upon native soil samples taken prior to the microcosm incubation setup (Basal), in order to assess possible treatment induced changes in soil chemistry when compared to the unamended parental substrate. After a 12-week incubation period, the soil chemical properties differed significantly between amended treatments (ANOSIM *R* = 0.872, *P* = 0.01, Supplementary Data), when referenced to the pre-incubation (Basal, dashed red line) and post-incubated (Control, dashed green line) chemical averages. Although all treatments were significantly different, no amendment Control and Nitrogen addition tended to be the less similar (ANOSIM *R* = 0.481, *P* = 0.01). One key environmental parameter, soil pH, remained constant across the experimental treatments, except for topsoil initially amended with the SMI (Inoculum), where pH significantly decreased from Basal levels of 7.74 to 7.13 when the SMI was added (*P* ≤ 0.05) (Figure 1 and Supplementary Table 1).

Iron (Fe), Copper (Cu), and Manganese (Mn) concentrations were significantly higher (*P* ≤ 0.05) in topsoil initially amended with the SMI (Inoculum) when compared to Basal, Control and Nitrogen treatments, likely as a result of growth media carry over within treatments amended with SMIs. Iron concentrations within the Microbes+Nitrogen (and to a lesser extent within the Microbes treatment) were also similar to the ones observed within the topsoil amended with SMIs. Ammonium Nitrogen (NH_4_-Nitrogen) was significantly higher (*P* ≤ 0.05) within the Nitrogen, Microbes, and Microbes+Nitrogen treatments when compared to Control, whilst NO_x_-N was only significantly higher (*P* ≤ 0.05) within the Microbes+Nitrogen treatment.

### Plant Growth Parameters

**FIGURE 2:**
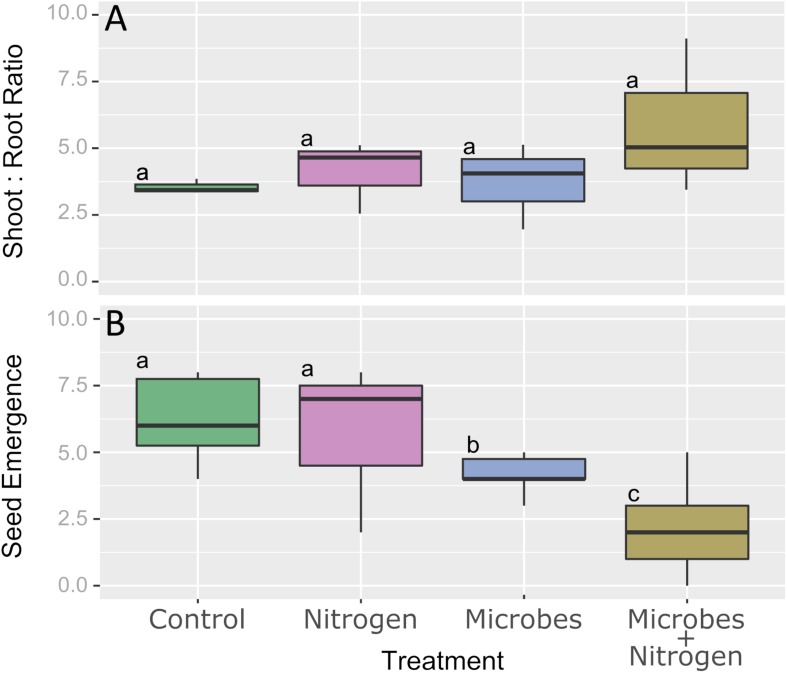
Boxplot representing **(A)** shoot:root ratio and **(B)** seed emergence across all 4 treatments. Boxplot sharing same letter coding are significantly similar (*P* ≤ 0.05).

After 12 weeks of topsoil incubation under the different treatments, no significant difference in calculated shoot:root ratio was observed (Figure 2, *P* ≤ 0.05). In terms of seedling emergence, the Control and Nitrogen only amendments were found to not be significantly different (*P* > 0.05), with an average of 6.3 and 5.9 seeds emerging for the Control and Nitrogen treatments, respectively (Figure 2B). In contrast, seedling emergence significantly decreased (*P* ≤ 0.05) when the SMIs were added. The addition of the SMI alone (Microbes) significantly reduced the emergence from an average of ca. 6 seeds per pot to around 3.5 seeds per pot (Figure 2B). The addition of nitrogen with the SMI (Microbes+Nitrogen) reduced the emergence success even further, to an average of around 2 seeds per pot to yield the lowest plant emergence over the whole experiment.

### Soil Microbial Activity

**FIGURE 3:**
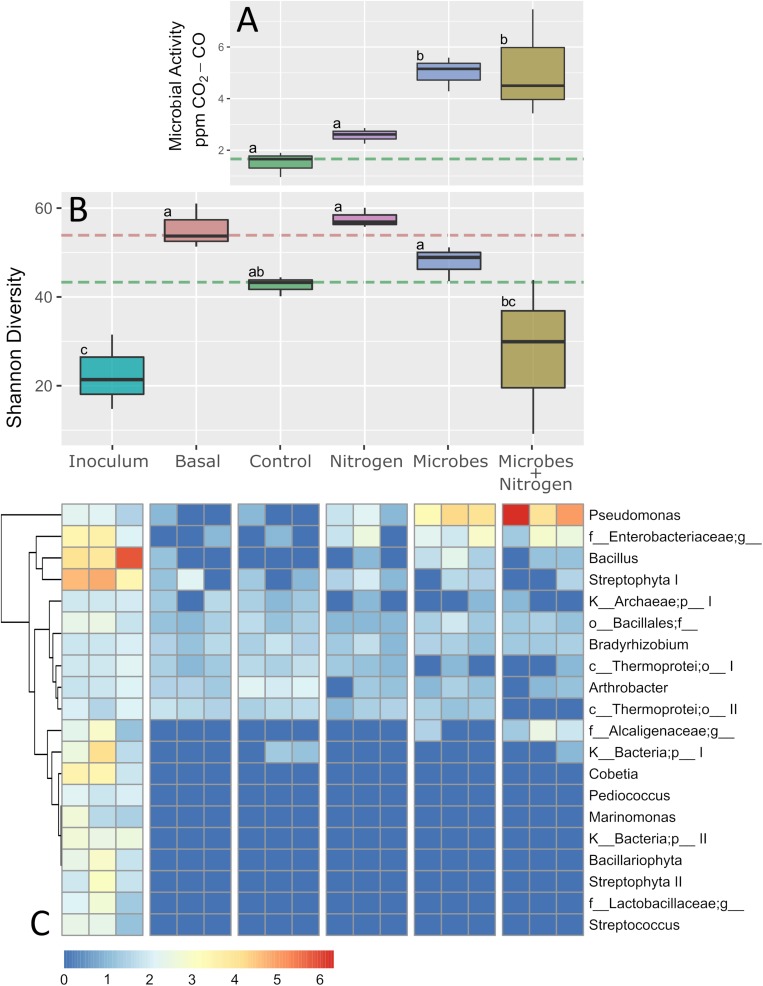
Composite figure showing microbial response, at physiological, ecological, and compositional levels, to treatments used in this study. **(A)** Shows microbial physiological activity, proxied as soil respiration changes. **(B)** Denotes Shannon diversity index of samples including SMI and pre-incubated levels (treatment Basal). Orange and green segmented lines represent basal and controls levels as per in Figure 1. **(C)** Heatmap represent the top-20 most abundant OTUs found in SMI samples as a way to track-down the fate of those allochthonous microorganisms in a semi-arid ecosystem. Boxplot sharing same letter coding are significantly similar (*p* ≤ 0.05).

Soil microbiome activity, assessed by CO_2_ evolution, significantly increased under the Microbes and Microbes+Nitrogen treatments (*P* ≤ 0.05 for both, Figure 3A) but not under the Nitrogen treatment (*P* > 0.05). Specifically, unamended controls and those with nitrogen only amendments evolved, on average, between 1.5 and 2.5 ppm-CO_2_, and were not significantly different from each other, indicating little stimulation of nascent microbial activity when nitrogen was added alone. However, when SMIs were added, both alone (Microbes) or with nitrogen (Microbes+Nitrogen) respiration significantly increased, with 5.13 and 5.0 ppm-CO_2_ being produced by the Microbes+Nitrogen and Microbes treatments, respectively (*P* ≤ 0.05, Figure 3A), representing at least a doubling of CO_2_ evolution over Control and Nitrogen treatments. Specifically, SMI addition under both Microbes and Microbes + Nitrogen treatments yielded CO_2_ evolutions which were much higher than under no SMI amendments, but which were not significantly different from each other (*P* > 0.05), suggesting the inclusion of nitrogen with the SMI had little extra stimulatory effect upon respiration within the microbiome.

### Microbiome Community Diversity and Evenness and Their Drivers

After 12 weeks of incubation, the emergent microbial communities differed between treatments (Figure 3B), where topsoil with no amendments (Control) exhibited a reduced Shannon index (by 23%) when compared to the parent soil used to initiate the experiment (Basal), indicating a one fifth diversity reduction due purely to experimental incubation. Within the treatments alone, the Nitrogen or Microbes treatments were not significantly different, in terms of microbiome diversity, from each other or when compared to the Control treatment (*P* > 0.05, Figure 3B). However, the addition of the SMI and nitrogen together (Microbes+Nitrogen) resulted in a significant decline (*P* < 0.05) in total microbiome community diversity when compared to all other treatments (Figure 3B). These (and all other microbiome Shannon diversity index observations) were mirrored within data generated for community evenness across all the imposed treatments (Supplementary Figure 1), suggesting a significant reduction in community diversity, and evenness of the microbiome but only when the SMI was present with nitrogen as a co-amendment.

Increases in soil microbiome respiration were seemingly at odds with significant decreases in overall microbiome diversity and evenness for the Microbes+Nitrogen treatment, which displayed strong CO_2_ evolution (Figure 3A) but the lowest Shannon Diversity (Figure 3C). This reduction in diversity and evenness suggested one of two possible mechanisms; either strong selection within the nascent microbiome to form a reduced community diversity and evenness, or, emergent dominance of a part of the added SMI.

In order to resolve which outcome was driving the observed community diversity, we compared the SMI microbiome diversity with those found within the treatments (Figure 3C). Microbiome sequence analysis of the SMI and in comparison, to the imposed treatments revealed that for the top 20 identified SMI taxa, only half of the taxa detected within the SMI could be reliably detected within the majority of the treatments tested (Figure 3C). For example, OTUs identified as *Marinomonas*, *Pediococcus*, *Streptococcus*, and one unknown genera within *Lactobacillaceae* were present within the SMI at appreciable abundances but were absent from all treatments tested after 12 weeks. SMI members that could be detected at varying levels within the treatments included a *Bacillales*, *Bradyrhizobium*, *and Arthrobacter* (Figure 3C). Interestingly, an unclassified member of the *Alcaligenaceae* was present in only the SMI and Microbes+Nitrogen, suggesting a likely stimulatory effect of nitrogen upon this SMI member.

When examining the specific differences across the treatments in relation to SMI addition with or without nitrogen, *Pseudomonas*, *Bacillus*, and one unclassified *Enterobacteriaceae* were the three SMI constituents which became abundant within the Microbes and Microbes +Nitrogen treatments (Figure 3C). Both the *Bacillus* and *Enterobacteriaceae* SMI members were relatively abundant within the SMI at the experimental initiation, whereas the *Pseudomonas* increased substantially in abundance in the Microbes treatment, and even higher within the Microbes+Nitrogen treatment when compared to its relative abundance in the original SMI. In fact, the *Pseudomonas* component became the most abundant OTU across the entire dataset within the Microbes+Nitrogen treatment (Figure 3C), suggesting this taxon grew strongly, and is likely the reason for the drop in diversity and evenness index within the Microbes+Nitrogen treatment (Figure 3B).

**FIGURE 4:**
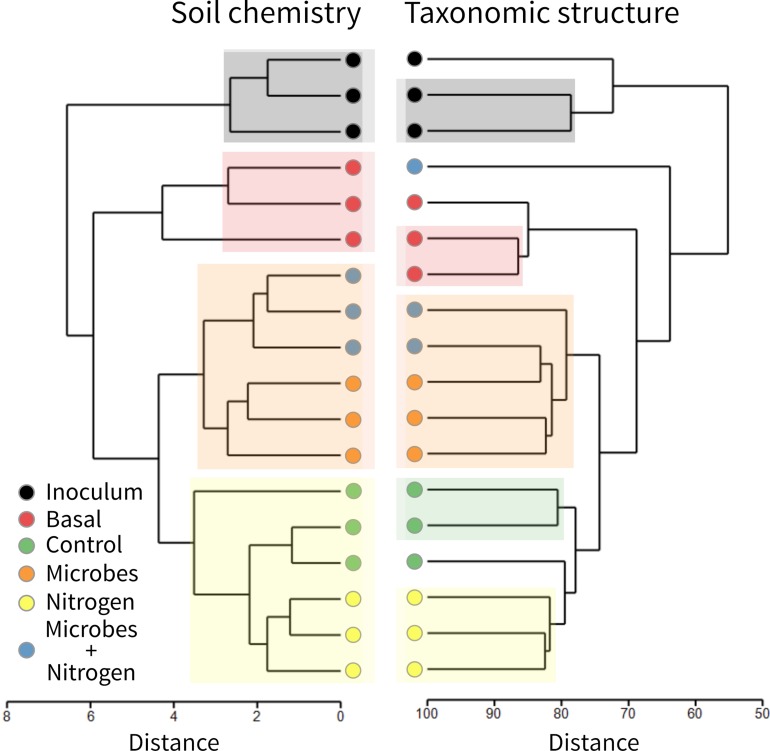
Hierarchical cluster analysis with SIMPROF test of soil chemistry **(left)** and 16S amplicon sequencing **(right)**. Colorized boxes represent similar groups founded with SIMPROF test.

**FIGURE 5:**
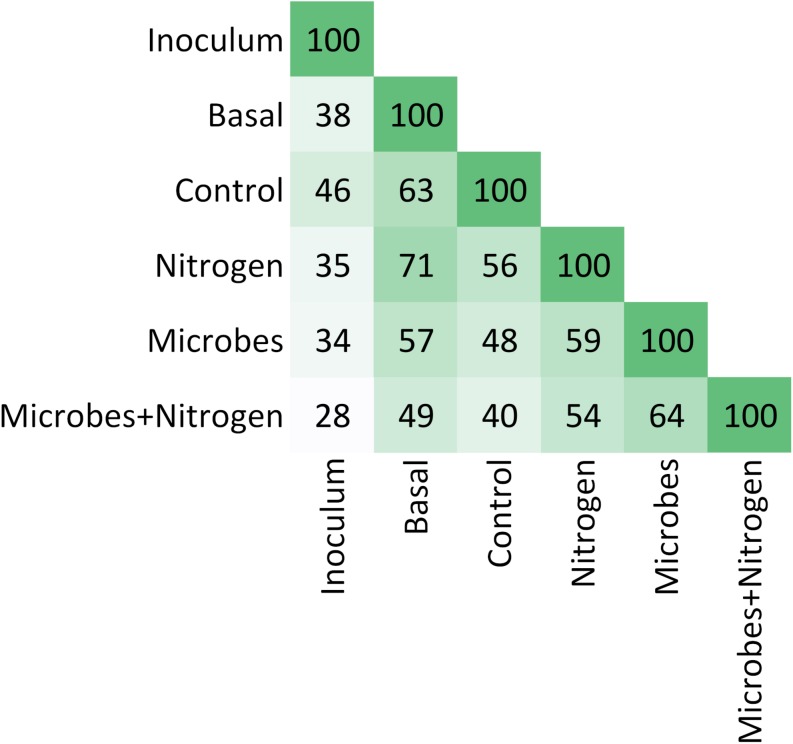
Pairwise heatmap showing OTUs similarities between treatments. See section “Discussion.”

In general terms, the OTU structure followed the same trend as that of that chemistry (Figure 4), in terms of similarity between treatments (Rand Index = 0.87), suggesting that soil chemistry played a central role in structuring the topsoil’s developing microbial community. SIMPROF identified 4 clusters within the soil chemistry and 5 clusters and 3 outgroups in the taxonomic table (at an order level). To examine the microbial diversity outcome of the added SMI, we searched for the OTUs found within treatment Inoculum across the entire dataset as a way to quantify treatment resemblance against it (Figure 5). The OTUs representing the SMI only composed a fraction of the total OTU pool obtained over all treatments, overlapping by 34 and 28% with treatment Microbes and Microbes+Nitrogen, respectively. Interestingly, OTU resemblance between the parent material (Basal) and treatment Nitrogen was as high as 71% shared OTUs, further demonstrating the recovery of post-incubated topsoil’s alpha diversity to the basal level’s when supplementing it with ammonium-sulfate (Figure 3B).

**FIGURE 6:**
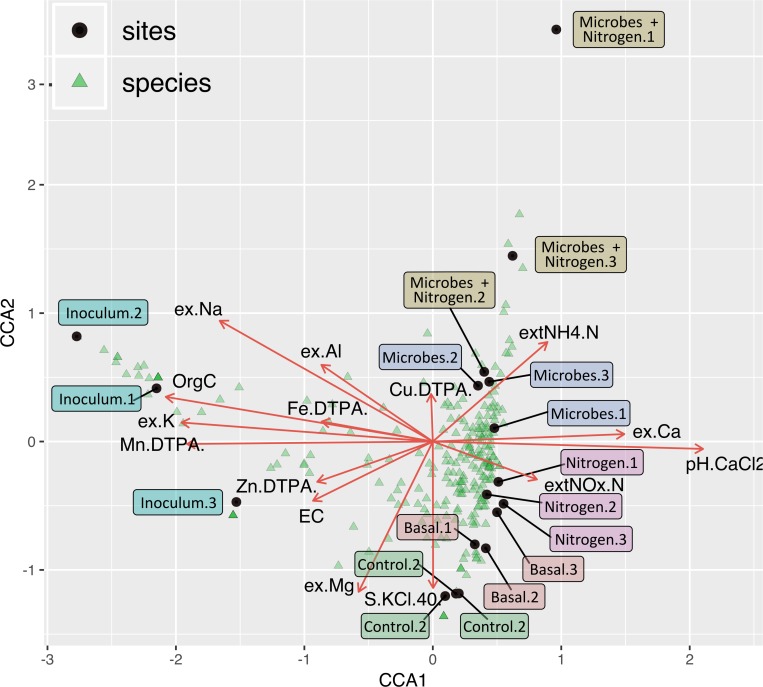
Correspondence analysis tri-plot showing soils samples (orange), taxonomic bins (at an order level, light blue), and soil chemistry parameters (brown arrow). While some orders are specific to certain treatments, a major part of the identified genera within the dataset are been shared by treatments control, basal, nitrogen, and microbes.

In order to link soil chemistry and taxonomic relative abundance canonical correspondence analysis (CCA) was computed (Figure 6). Taxonomic bins (Order level, light blue triangles) were well scattered across treatments, albeit several orders were situated around the plot center, denoting a shared microbiome across all 4 treatments and with the parent material (Basal). However, SMI addition caused these treatments to cluster apart with the taxonomic diversity within Microbes and Microbes+Nitrogen being explained by the soil concentration of NH_4_-N whilst taxonomic variability within the Nitrogen treatment was explained by pH and Ca (Figure 6).

## Discussion

We studied the effect of a commercially available soil microbial inoculum (SMI) within a mine site, semi-arid ecosystem context. We assessed soil microbiome and plant parameters as a first approach to gauge whether currently available SMIs can be applied to mine site restoration practices to enhance aboveground outcomes and overcome the reduced microbiome capacity within soils which have been subjected to mineral extraction (Kumaresan et al., 2017). We further tracked the most abundant OTUs from the SMI to understand if such microorganisms can survive the relatively harsh conditions within mine waste soil for subsequent planting of native flora.

Treatment selection in this experiment (Control, Nitrogen, Microbes, and Microbes+Nitrogen) can be viewed as an increasing intervention scale within the topsoil’s native microbiome and the soil’s chemical properties. The nitrogen addition treatment did not contain any allochthonous microorganisms and the differences in microbial diversity, evenness, or soil respiration shown in this study were similar to the experimental controls. However, the addition of the SMI, principally under the Microbes+Nitrogen treatment, substantially altered the plant response, reducing seedling emergence, microbial activity, and overall taxonomic diversity. Since the SMI was derived from agricultural soils and used in a more depauperate ecosystems we conclude that reduction in seedling emergence is likely due to a mismatch between plant and the introduced microbiome and that key drivers of emergence are likely present in the native microbiome associated with a given species of plant.

These observations are in line with previous suggestions that native microbial communities drive aboveground diversity (Wagg et al., 2014; Wubs et al., 2016; van der Putten, 2017), improve plant overall fitness (Lau and Lennon, 2012), dictate seed bank persistence [reviewed in Long et al. (2015)], or even affecting flowering time (Panke-Buisse et al., 2015). Similar to our results, Batten et al. (2008) demonstrated that changes in the soil indigenous microbiome (in this case, mediated by invasive plants) negatively affected the performance of the American native plant *Lasthenia californica*. The net effects of soil microbial symbionts upon plant fitness can fluctuate along the mutualism-parasitism continuum depending on their origin, genetics, and environmental conditions (Klironomos, 2003; Denison and Kiers, 2004). Hence, the reduced seedling emergence in the presence of the SMI is likely associated with the (a) reduced abundance and diversity of the indigenous microbes that presumably promote seed germination/emergence, and/or (b) negative/incompatible interactions between the native plant *A. ancistrocarpa* and the microorganisms introduced via addition of the microbial consortium. Clearly, further work investigating native plant and microbe signaling will be crucial to unveil such key constraints to seed emergence within these semi-arid ecosystems.

### SMI With or Without Nitrogen Addition Does Not Improve Plant Fitness

The proposed key linkages between the native microbial diversity and plant fitness (germination, biomass) were evident when considering a decline of 33% in seedling emergence was observed when SMIs were added with Nitrogen, when compared to the Control. This implied that a severe loss in the topsoils’ native microbiome (as observed for SMI addition treatments) could significantly impact plant recruitment. Currently, we cannot fully explain the exact mechanism, other than highlight there must be key linkages for this species to the native microbiome, as our knowledge is limited on both biological entities (i.e., *A. ancistrocarpa* seedlings and native microbiome). However, it is widely accepted that the soil microbiome is integral for seed germination, as it can mediate seed coat break down (van Leeuwen, 1981; Delgado-Sánchez et al., 2011), degrade germination inhibitors (Zhu et al., 2011), and/or protect the seeds from pathogenic attack (Dalling et al., 2011). As discussed above, a fraction of the reduced plant fitness could be linked with incompatible/pathogenic interactions between the exogenous microbes and the native plant. Furthermore, it has been shown that seed exudates can drive microbiome composition in the immediate surroundings, either by encouraging microbial growth via the supply of carbon compounds (Roberts et al., 2009) or discouraging them by the production of defense proteins (Rose et al., 2006) such as peroxidases (Fuerst et al., 2011). We await the resolution of the detailed mechanisms in this case, but in general, a dense beneficial microbial assemblage is often formed around seeds (Chee-Sanford et al., 2006) and clearly altering such an assemblage for native semi-arid species, as here, has significant repercussions for seed success.

Depletion of the initial native microbiome was seen throughout the incubation experiment when comparing the characteristics of the parental topsoil (Basal) and the Control incubation treatment. This loss in both diversity and evenness over time within the control soil is a parallel for topsoil storage scenarios and is in line with previous studies documenting decreases in archaeal and bacterial microbiome diversity, functional capacity (Kumaresan et al., 2017), earthworm communities (Boyer et al., 2011), and seedling recruitment (Rokich et al., 2000; Golos and Dixon, 2014) when fresh topsoil is stored unplanted for later use. We observed significant decreases in microbiome diversity and evenness (up to 20%) even in a relatively short 12-week time period. The impact of the loss of microbiome diversity during storage/experimental incubation must be assessed with caution, however, as higher microbiome diversity does not always correlate with healthier ecosystems (Fernandez et al., 2000; Hashsham et al., 2000). Shade (2017) argue that comparing microbiomes by their diversity indices and assuming that higher diversity is better, oversimplifies the underlying mechanisms that set such diversities values. Nevertheless, more diverse microbial communities signify a higher potential for metabolic redundancy and, by extension, community resilience and plasticity. Despite clear changes within the native microbiomes during storage/incubation, stockpiling is a common practice in open cut mines where topsoil is removed from its original site, and stored elsewhere (Muñoz-Rojas et al., 2016a) in conditions that do not resemble its natural planted state. While this practice seems to be a logical procedure for restoration, a priority must be the resolution of whether better storage protocols can improve the conservation of the native belowground microbial communities (Shade, 2017) and, by extension, improve the success of above and belowground outcomes when restoration commences.

### SMI OTUs Have Differential Response to Semi-Arid Conditions

Many component taxa within the SMI clearly found the semi-arid conditions challenging for establishment. Despite the ease of access to commercial SMIs, they tend to be derived from high nutrient agricultural systems, systems which are vastly different from the edaphic conditions within semi-arid soils. The last can be explained by the differential interest from both the academia and the industry to develop novel ways to enhance soil quality (O’Callaghan, 2016; Muñoz-Rojas, 2018).

Several criteria include substantial differences in water and nutrient availability, metal concentrations and physical structure. Here, from the 40 most abundant OTUs within the SMI, 16 could not be detected reliably within the treatments, and 12 OTUs were present across all the treatments. Amongst these, four fell within the order *Rhizobiales* (*Microvirga*, *Bradyrhizobium*, an unknown family and an unknown genus within *Hypomicrobiaceae*). Such microorganisms may play a fundamental role in the early stages of primary succession (Van Der Heijden et al., 2008; Wubs et al., 2016), as they can be the only source of nitrogen in such nutrient-limited soils. Specifically, such organisms have been demonstrated within early ecological succession of glacier forefronts (Knelman et al., 2012), where a strong correlation between similar OTUs with vegetation development has been observed (King et al., 2010). Addition of symbiotic or free-living nitrogen fixing microorganisms to semi-arid environment can be a significant step to potentiate nitrogen cycle in mine-impacted soils, especially addition of free-living diazotrophs, which has been shown to be active N_2_ fixers (Buckley et al., 2007) in many terrestrial systems (Cleveland et al., 1999). *Hypomicrobiaceae* members are of great interest as they are ubiquitous soil microorganisms (Oren and Xu, 2014) known to be abundant in both mining impacted environments (Reis et al., 2013) and in agricultural soils (Zhalnina et al., 2013), which opens the possibility for future approaches using them to initiate N cycling dynamics. An abundant OTU falling within the *Arthrobacter* genus was also found across all treatments, where *Arthrobacter* spp. are among the most ubiquitous indigenous soil bacteria that harbor broad metabolic and ecological functions to cope with harsh conditions (Mongodin et al., 2006), and have been detected as plant endophytes (Hardoim et al., 2015), within soda lakes (Duckworth et al., 1996; Chakkiath et al., 2013), and mine tailings (Bondici et al., 2013). Microorganisms with such ubiquity and functional plasticity can be suitable components for early colonization of nutrient-limited, semi-arid environments and post-mining vegetation rehabilitation.

## Conclusion

In this study, we analyzed changes occurring within mine site topsoil microbiomes and chemical parameters after a 12-week incubation experiments using agriculture-based SMI and/or ammonium sulfate. This is the first study to assess the efficacy of protocol translation from agriculture practices into a semi-arid restoration context, shedding light into an establishing, but poorly studied restoration practice. Our results revealed that an important depletion of semi-arid microbiome diversity and evenness occurred when SMIs were added and further exacerbated when ammonium sulfate was also added in conjunction. Such a loss in native microbial diversity, along with incompatible interactions from exogenous microbes, likely explained the loss in *A. ancistrocarpa* fitness proxies (seedling emergence and shoot to root ratio). Therefore, future mine site restoration protocols must carefully consider preservation of native microbiome diversity through appropriate topsoil handling and storage as well as careful selection of any exogenous taxa that may be added to maximize a protocol’s potential success. For the latter, key taxa which deliver primary ecosystem functions (such as N fixation) and can survive the semi-arid conditions are a priority. Here, we demonstrate that α-proteobacterial nitrogen fixing organisms are likely to be of particular interest and suitability to speed-up nitrogen cycle restoration in mining affected areas.

## Author Contributions

MM-R and DK designed the glasshouse trial. BM-G, MM-R, KK, and DK performed the experiments. BM-G, MM-R, and DK analyzed the data. BM-G wrote the first draft with significant inputs on subsequent drafts from AW and AO’D. PS facilitated the SMI and consulted throughout the study. All authors reviewed and approved the final version of this manuscript.

## Conflict of Interest Statement

PS serves as Director of R&D, Troforte Innovations Pty Ltd., a company which sells the SMI used in this experiment. The remaining authors declare that the research was conducted in the absence of any commercial or financial relationships that could be construed as a potential conflict of interest.
