# Supplementary material for: Reconditioning Degraded Mine Site Soils With Exogenous Soil Microbes: Plant Fitness and Soil Microbiome Outcomes
Source: Front Microbiol. 2019 Jul 10;10:1617. doi: 10.3389/fmicb.2019.01617 (PMC6636552; doi:10.3389/fmicb.2019.01617)
Supplement: Supplementary file 2 [file Data_Sheet_2.docx]

**Supplementary table and figure**

|  | **pH** | | **EC** | | **Al** | | **Ca** | | **K** | | **Mg** | | **Na** | | **S** | | **Cu** | | **Fe** | | **Mn** | | **Zn** | | **NH4-N** | | **NOx-N** | | **Org-C** | |
| --- | --- | --- | --- | --- | --- | --- | --- | --- | --- | --- | --- | --- | --- | --- | --- | --- | --- | --- | --- | --- | --- | --- | --- | --- | --- | --- | --- | --- | --- | --- |
|  | CaCl_2_ | | dS/m | | ex | | ex | | ex | | ex | | ex | | KCl-40 | | DTPA | | DTPA | | DTPA | | DTPA | | ex | | ex | | mg/Kg |  |
| **Inoculum** | 7.13 | d | 279.33 | a | 0.10 | a | 8.74 | c | 2.00 | c | 1.88 | b | 0.53 | a | 51.83 | b | 1.08 | c | 7.28 | a | 17.03 | a | 0.99 | a | 6.00 | c | 12.33 | ed | 1.41 | b |
| S.D. | 0.06 |  | 75.08 |  | 0.03 |  | 0.21 |  | 0.18 |  | 0.10 |  | 0.08 |  | 7.99 |  | 0.02 |  | 0.89 |  | 1.03 |  | 0.02 |  | 0.00 |  | 5.51 |  | 0.16 |  |
|  |  |  |  |  |  |  |  |  |  |  |  |  |  |  |  |  |  |  |  |  |  |  |  |  |  |  |  |  |  |  |
| **Basal** | 7.74 | ab | 150.33 | a | 0.05 | cd | 9.84 | ab | 0.51 | c | 1.86 | b | 0.33 | b | 32.33 | b | 0.96 | d | 3.07 | c | 6.07 | d | 0.90 | ab | 7.30 | bc | 10.50 | e | 0.89 | a |
| S.D. | 0.04 |  | 31.63 |  | 0.01 |  | 0.25 |  | 0.02 |  | 0.04 |  | 0.01 |  | 12.84 |  | 0.06 |  | 0.21 |  | 0.55 |  | 0.17 |  | 1.70 |  | 3.41 |  | 0.04 |  |
|  |  |  |  |  |  |  |  |  |  |  |  |  |  |  |  |  |  |  |  |  |  |  |  |  |  |  |  |  |  |  |
| **Control** | 7.77 | ab | 299.67 | a | 0.03 | d | 10.10 | ab | 0.50 | c | 2.20 | a | 0.13 | c | 242.43 | a | 0.82 | e | 2.90 | c | 7.23 | c | 0.80 | b | 4.20 | c | 20.00 | bc | 0.90 | a |
| S.D. | 0.06 |  | 205.83 |  | 0.01 |  | 0.44 |  | 0.05 |  | 0.14 |  | 0.01 |  | 19.61 |  | 0.02 |  | 0.00 |  | 0.32 |  | 0.10 |  | 3.12 |  | 0.70 |  | 0.05 |  |
|  |  |  |  |  |  |  |  |  |  |  |  |  |  |  |  |  |  |  |  |  |  |  |  |  |  |  |  |  |  |  |
| **Nitrogen** | 7.81 | a | 154.20 | a | 0.07 | bc | 10.33 | a | 0.37 | c | 1.64 | c | 0.31 | b | 31.53 | b | 0.97 | d | 3.27 | c | 5.70 | d | 0.80 | b | 12.00 | a | 16.63 | cd | 0.91 | a |
| S.D. | 0.04 |  | 3.83 |  | 0.02 |  | 0.25 |  | 0.04 |  | 0.05 |  | 0.02 |  | 8.89 |  | 0.02 |  | 0.38 |  | 0.26 |  | 0.00 |  | 2.96 |  | 3.67 |  | 0.02 |  |
|  |  |  |  |  |  |  |  |  |  |  |  |  |  |  |  |  |  |  |  |  |  |  |  |  |  |  |  |  |  |  |
| **Microbes** | 7.72 | bc | 197.10 | a | 0.10 | ab | 9.73 | ab | 0.98 | b | 1.81 | b | 0.30 | b | 45.20 | b | 1.19 | b | 6.27 | b | 9.13 | b | 0.87 | ab | 11.00 | ab | 23.17 | b | 0.93 | a |
| S.D. | 0.06 |  | 9.98 |  | 0.03 |  | 0.67 |  | 0.01 |  | 0.10 |  | 0.04 |  | 21.75 |  | 0.08 |  | 0.21 |  | 0.23 |  | 0.06 |  | 2.08 |  | 2.75 |  | 0.01 |  |
|  |  |  |  |  |  |  |  |  |  |  |  |  |  |  |  |  |  |  |  |  |  |  |  |  |  |  |  |  |  |  |
| **Both** | 7.65 | c | 205.93 | a | 0.06 | cd | 9.43 | b | 0.95 | b | 1.60 | c | 0.27 | b | 41.77 | b | 1.28 | a | 7.87 | a | 10.03 | b | 0.97 | a | 13.23 | a | 39.10 | a | 1.00 | a |
| S.D. | 0.00 |  | 2.51 |  | 0.01 |  | 0.30 |  | 0.01 |  | 0.06 |  | 0.02 |  | 16.74 |  | 0.03 |  | 0.97 |  | 0.12 |  | 0.06 |  | 3.88 |  | 2.57 |  | 0.03 |  |

Supplementary table 1. Soil chemistry mean parameters and standard deviation (S.D.) across different treatments. Means values followed by different letters denotes they are significantly different (P ≤ 0.05)


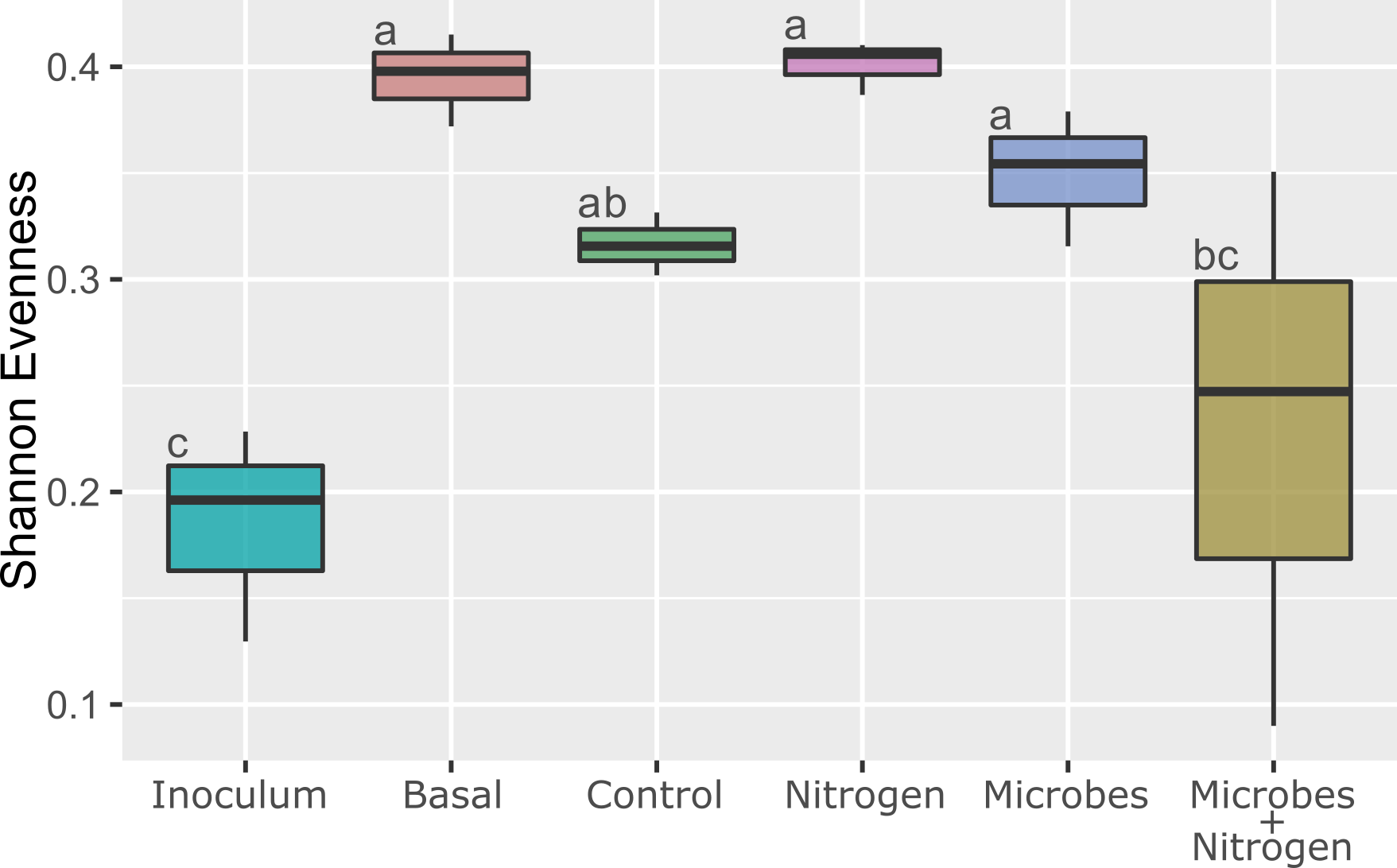


Supplementary fig. 1. Shannon evenness boxplot based on the OTU table at an order level. Boxplots sharing same letters represents so significant differences (P ≤ 0.05)
